# Third Generation Cephalosporin Resistant *Enterobacterales* Infections in Hospitalized Horses and Donkeys: A Case–Case–Control Analysis

**DOI:** 10.3390/antibiotics10020155

**Published:** 2021-02-04

**Authors:** Anat Shnaiderman-Torban, Dror Marchaim, Shiri Navon-Venezia, Ori Lubrani, Yossi Paitan, Haya Arielly, Amir Steinman

**Affiliations:** 1 Koret School of Veterinary Medicine (KSVM), The Robert H. Smith Faculty of Agriculture, Food and Environment, The Hebrew University of Jerusalem, Rehovot 7610001, Israel; ashnaiderman@gmail.com (A.S.-T.); ori.lubrani@mail.huji.ac.il (O.L.); 2 Unit of Infection Control, Shamir (Assaf Harofeh) Medical Center, Zerifin, Beer Yaakov 70300, Israel; drormarchaim@shamir.gov.il; 3 Sackler School of Medicine, Tel-Aviv University, Tel-Aviv 69978, Israel; 4 Department of Molecular Biology, Faculty of Natural Science, Ariel University, Ariel 40700, Israel; shirinv@ariel.ac.il; 5 The Miriam and Sheldon Adelson School of Medicine, Ariel University, Ariel 40700, Israel; 6 Department of Clinical Microbiology and Immunology, Sackler Faculty of Medicine, Tel Aviv University, Tel Aviv 6997801, Israel; yossi.paitan@clalit.org.il (Y.P.); Ariellyhaya@clalit.org.il (H.A.); 7 Clinical Microbiology Lab, Meir Medical Center, Kfar Saba 4428164, Israel

**Keywords:** cephalosporins, extended-spectrum β-lactamase, equine, resistance, case–case–control

## Abstract

In human medicine, infections caused by third-generation cephalosporin-resistant *Enterobacterales* (3GCRE) are associated with detrimental outcomes. In veterinary medicine, controlled epidemiological analyses are lacking. A matched case–case–control investigation (1:1:1 ratio) was conducted in a large veterinary hospital (2017–2019). In total, 29 infected horses and donkeys were matched to 29 animals with third-generation cephalosporin-susceptible *Enterobacterales* (3GCSE) infections, and 29 uninfected controls (overall *n* = 87). Despite multiple significant associations per bivariable analyses, the only independent predictor for 3GCRE infection was recent exposure to antibiotics (adjusted odds ratio (aOR) = 104, *p* < 0.001), but this was also an independent predictor for 3GCSE infection (aOR = 22, *p* < 0.001), though the correlation with 3GCRE was significantly stronger (aOR = 9.3, *p* = 0.04). In separated multivariable outcome models, 3GCRE infections were independently associated with reduced clinical cure rates (aOR = 6.84, *p* = 0.003) and with 90 days mortality (aOR = 3.6, *p* = 0.003). *Klebsiella* spp. were the most common 3GCRE (36%), and *bla*_CTX-M-1_ was the major β-lactamase (79%). Polyclonality and multiple sequence types were evident among all *Enterobacterales* (e.g., *Klebsiella pneumoniae*, *Escherichia coli*, *Enterobacter cloacae*). The study substantiates the significance of 3GCRE infections in equine medicine, and their independent detrimental impact on cure rates and mortality. Multiple *Enterobacterales* genera, subtypes, clones and mechanisms of resistance are prevalent among horses and donkeys with 3GCRE infections.

## 1. Introduction

Third-generation cephalosporin-resistant *Enterobacterales* (3GCRE) are spreading worldwide [1]. Resistance is mainly due to the production of plasmid-mediated extended-spectrum β-lactamases (ESBLs) and AmpC β-lactamases, as well as the hyper-production of chromosomal Amp-C β-lactamases [2]. In human medicine, infections caused by 3GCRE are often associated with a delay in the initiation of appropriate antibiotic therapy, and therefore with worse clinical outcomes [3], since delays in the initiation of appropriate therapy are the strongest modifiable independent predictor for mortality in adult inpatients with severe sepsis [4]. In well-designed analyses in humans, these infections were independently associated with higher mortality rates, increased hospital charges, and longer lengths of hospital stay (LOS) [3]. This was further demonstrated in high-risk human patients, where infection with ESBL-producing *Enterobacterales* (ESBL-PE) has been shown to affect the clinical outcome by leading to an increased rate of inadequate initial therapy and a higher mortality [5]. A major concern regarding 3GCRE infections, and specifically ESBL-PE infections, is co-resistances to additional classes of therapeutical options, i.e., fluoroquinolones, aminoglycosides, trimethoprim-sulfamethoxazole. This further contributes to the epidemiological significance of these infections, both in human and in veterinary medicine [6,7].

Third-generation cephalosporins are critically important veterinary antimicrobials, as defined by the World Organization for Animal Health [8]. However, in recent years, there have been increasing reports pertaining to colonization and infections caused by 3GCRE among animals [9]. In equine medicine, reports of 3GCRE and in particular ESBL-PE infections are emerging, both in the community and in healthcare settings [10]. Shedding rates of 3GCRE by healthy horses in farms were reported worldwide, varying from 5.2% to 44% [11,12,13,14,15]. In three different studies, conducted in two different equine hospitals, shedding rates were shown to increase by 2.5–5.1-fold during hospitalization, implying that the nosocomial acquisition and spread of these resistant bacteria is common in certain veterinary facilities [11,16,17]. Moreover, there are numerous reports on various severe and invasive 3GCRE infectious syndromes among horses, e.g., skin and soft tissue infections, surgical site infections, upper respiratory tract infections, and bacteremia [18,19,20,21]. Furthermore, in horses, synovial infection with multidrug-resistant (MDR) bacteria was significantly associated with euthanasia [22]. However, the controlled scientific evidence, pertaining to risk factors and outcomes, which are independently associated with 3GCRE infections in equine medicine, is scarce.

In human medicine, the case–case–control methodology is considered today the “gold standard” in terms of analyzing risk factors/predictors in the field of antimicrobial resistance [23]. In this nested matched case-control design, every patient with a resistant pathogen is matched to a patient with a susceptible pathogen and to a patient with no pathogen (i.e., uninfected control). This methodology enables us to point out the specific predictors independently associated with the resistance determinant, while "diluting" the impact of the infection itself (i.e., by either a resistant or a susceptible strain). In veterinary medicine, as far as we know, there are no reported case–case–control studies in the field of antimicrobial resistance among animals. Our study aims were to conduct a matched case–case–control investigation, to study the predictors and outcomes, which are independently associated with 3GCRE infections among horses and donkeys.

## 2. Results

### 2.1. Population Characteristics

During the study period, there were 1564 admissions of horses and 56 admissions of donkeys recorded at the Koret School of Veterinary Medicine, Veterinary Teaching Hospital (KSVM-VTH) (Table S1). Overall, 232 clinical specimens were submitted to the bacteriological lab, of which 32 specimens (14%), which were obtained from 29 animals, grew 3GCRE. The 29 patients with 3GCRE infection (“resistant cases”) were then matched to 29 patients with third-generation cephalosporin-susceptible *Enterobacterales* (3GCSE) infection (“susceptible cases”), and to 29 patients with no infection ("uninfected controls"). In total, 87 animals were enrolled (82 horses and 5 donkeys). The median age of the entire cohort was 2.75 years (range 0–24), the main breed was Arabian (48.3%, *n* = 42/87), 2.3% were geriatric (*n* = 2/87), 41.4% were neonates (*n* = 36/87), 10.3% were shelter residents (*n* = 9/87), 59.8% were females (*n* = 52/87), and out of 17 adult males, 64.7% were castrated (*n* = 11/17, i.e., 18 males were neonatal colts and were therefore not castrated and not included in the denominator for this calculation). Specifically for the donkeys, all five were adults, three were females and two were not castrated males. Eight percent (7/87) of all patients were hospitalized in the preceding three months, and the median length of stay was eight days (range: 2–181 days).

### 2.2. Predictors of 3GCRE Infections

Table 1 summarizes selected bivariable analyses conducted between the three groups of patients.

Table 1 depicts a summarization of the bivariable analyses conducted between the three study groups. Most predictors associated with a 3GCRE infection in bivariable analysis were also associated with 3GCSE infection, including recent surgeries, recent invasive procedures and recent exposure to multiple classes of antibiotics. In the multivariable matched model of patients with 3GCRE infection vs. uninfected controls, the only independent predictor remaining in the model was recent exposure to antibiotics (adjusted odds ratio (aOR) = 104, 95% CI 9.778–1106.182, *p* < 0.001). However, recent exposure to antibiotics remained also the only predictor associated with 3GCSE infection (aOR = 22, 95% CI 5.086–92.303, *p* < 0.001). In a matched multivariable model of patients with 3GCRE infection vs. patients with 3GCSE infection, recent exposure to antibiotics was significantly and independently associated with 3GCRE infection (aOR = 9.3, 95% CI 1.06–80.934, *p* = 0.04).

### 2.3. Clinical Outcomes of 3GCRE Infections

In bivariable outcome analyses, 3GCRE infections were significantly associated with in-hospital mortality, 14-days mortality, 90-days mortality, 1-year mortality, upper airway procedure following the infection, surgery following the infection, and longer LOS (after excluding the patients who died), and was significantly associated with clinical failure (Table 2). In separate multivariable models for each of these variables, 3GCRE infection remained independently associated with failure of clinical cure (aOR = 6.84, 95% CI 1.919–24.39, *p* = 0.003), 90-days mortality (aOR = 3.623, 95% CI 1.107–11.863, *p* = 0.003) and with surgery following the infection (aOR = 3.364, 95% CI 1.169–9.685, *p* = 0.025). In a sub-analysis that included only patients with 3GCRE or 3GCSE infection, 3GCRE infection was independently and negatively associated with the administration of appropriate antibiotic therapy throughout the course of illness (OR = 0.041, 95% CI 0.009–0.187, *p* < 0.001), and in terms of the number of days for which the appropriate antimicrobials were administered (*p* < 0.001) (Table 2).

### 2.4. GCRE Samples Description, Species Distribution and Resistance Rates

**Figure 1:**
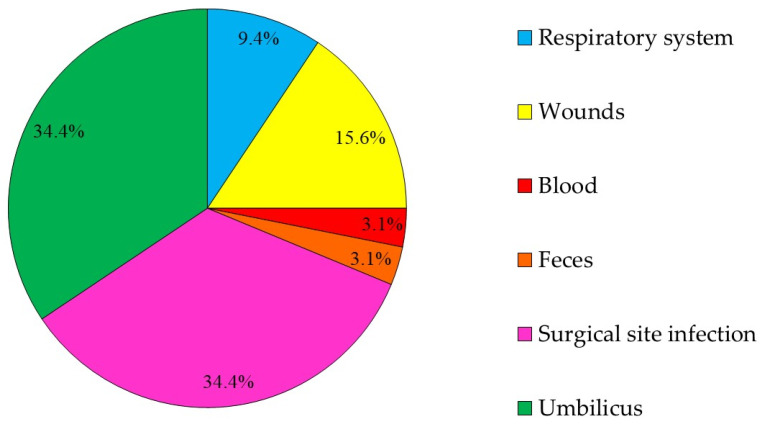
The distribution according to the source (body-site) from which the 3GCRE pathogen was isolated (*n* = 32 cultures).

**Figure 2:**
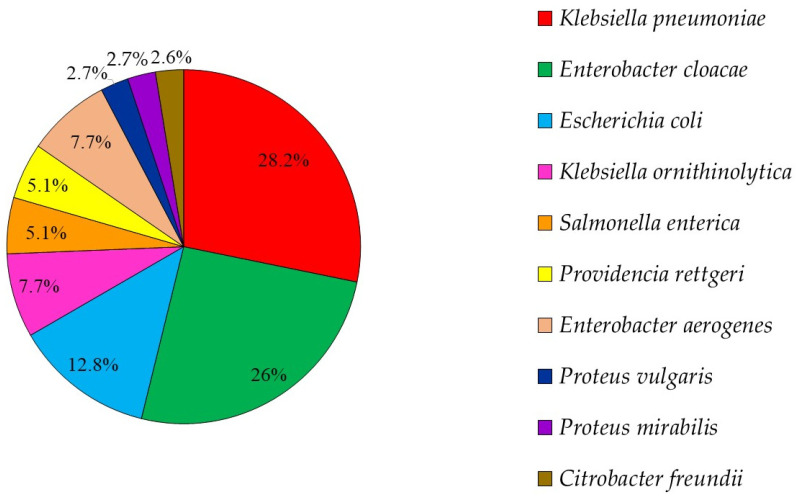
3GCRE species distribution (*n* = 39 pathogens).

**Figure 3:**
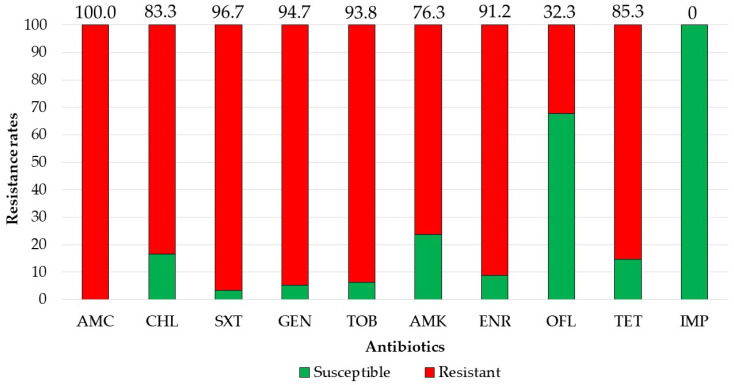
Resistance rates of 3GCRE pathogens (*n* = 39) towards commonly prescribed agents. AMC, amoxicillin-clavulonate; CHL, chloramphenicol; SXT, trimethoprim-sulfamethoxazole; GEN, gentamicin; TOB, tobramycin; AMK, amikacin; ENR, enrofloxacin; OFL, ofloxacin; TET, tetracycline; IMP, imipenem.

There were 39 3GCRE isolates, recovered from 32 clinical specimens, obtained from 29 patients. Twenty-one (65.6%) cultures were polymicrobial. Ten samples (31.25%) were collected from hospitalized equids during the first 48 hours of hospitalization, i.e., suggesting acquisition in the community [29]. The two most prevalent infectious syndromes were umbilical cord [30] and surgical-site infections (i.e., SSI; Figure 1). SSIs were following either laparotomy or orthopedic surgery (50% each). Of the 39 3GCRE isolates (Figure 2), the major pathogens were *Klebsiella* spp. (*n* = 14/39, 35.89%), *Enterobacter* spp. (*n* = 13/39, 33.33%), and *Escherichia coli* (*n* = 5/39, 12.82%). The resistance rates of the isolates to the commonly prescribed agents in veterinary medicine, in addition to β-lactams, are depicted in Figure 3. Nearly all isolates (38/39, 97.43%) were categorized as MDR organisms (MDRO) [24].

### 2.5. Molecular Characteristics of 3GCRE Isolates 

Of the 39 3GCRE isolates, 26 (66.67%) were identified as ESBL producers via phenotypic tests. Nineteen of those (*n* = 19/26, 73.1%) were available for further molecular analyses. Of those, 17 isolates (89.5%) were *bla*_CTX-M_ producers; i.e., the majority were *bla*_CTX-M-1_ (*n* = 15/17, 88.2%), followed by *bla*_CTX-M-9_ (*n* = 2/17, 11.8%).

The multi locus sequence type (MLST) of the three major species (*K. pneumoniae, E. cloacae*, *E. coli*) revealed the presence of polyclonality and diverse groups of sequence types (ST). The six *K. pneumoniae* isolates belonged to ST35 (umbilical infection, SSI—one isolate of each), ST13 (two isolates originated from umbilical infections), ST985 (one isolate from a wound), and ST528 (one isolate from an umbilicus). The three *E. coli* isolates were ST38 (blood), ST361 (umbilicus), and ST2179 (respiratory tract). The four *E. cloacae* were ST182 (wound), ST66 (respiratory tract), and ST254 and ST135 (umbilicus both).

## 3. Discussion

In 2016, the World Health Organization (WHO) declared infections resulting from MDRO to pose one of the major challenges and threats to humanity [31]. In equine medicine, the incidence of MDRO infections has risen exponentially in recent years [32], along with scrutiny, awareness and assessment for the proper usage of antimicrobials, infection control measures, the development of practice standards, and incorporating the routine use of clinical microbiology practices [33]. *Enterobacterales* are a major group of MDRO recognized by the WHO [31]. This group of pathogens became resistant to ESBL agents (i.e., 3GCRE), which are among the most common, efficacious and bactericidal antimicrobial agents. In order to implement established measures in infection control and antimicrobial stewardship (AMS), i.e., to curb the continued emergence and spread of these 3GCRE pathogens, detailed and controlled epidemiological analyses in veterinary hospitals are warranted. Therefore, a matched case–case–control investigation was executed in a large university-affiliated veterinary hospital, to explore the clinical and molecular epidemiology of 3GCRE infections among equids. The matched case–case–control design is considered today the “gold standard” methodology in investigating risk factors in the field of MDRO emergence and transmission. This design enables us to explore the independent predictors for the emergence of the MDRO, while controlling for multiple biases and confounders associated with “the infection” in general [23]. In order to tailor appropriately and implement a successful prevention strategy, a controlled analysis isolating the true independent predictors associated with the emergence of the resistance determinant per se is warranted.

In this case–case–control investigation, 29 animals (24 horses and 5 donkeys) with 3GCRE infection were matched to 29 animals with 3GCSE infections, and 29 uninfected controls (overall *n* = 87). In bivariable analyses (Table 1), there were multiple significant associations with 3GCRE infections, as compared to uninfected controls, e.g., recent hospitalizations, previous recent invasive procedures, plasma therapy, and recent exposure to antibiotics (specifically to penicillins, fluoroquinolones, aminoglycosides and polymyxins). In multivariable analysis, only exposure to antimicrobials remained an independent predictor of 3GCRE infection. However, in the multivariable model of 3GCSE infections vs. uninfected controls, exposure to antimicrobials was also the only independent predictor of 3GCSE infection, implying this is a predictor for infection in general, not a predictor for the emergence or acquisition of the resistance determinant. It must be noted though that the association with recent exposure to antimicrobials was much stronger among the 3GCRE group, and in a multivariable model of patients with 3GCRE infection vs. patients with 3GCSE infection, exposure to antimicrobials was an independent predictor for 3GCRE infection (aOR = 9.3, 95% CI 1.1–81).

In general, there are two modes by which an animal could acquire an MDRO: (1) patient-to-patient transmission (e.g., from another animal, through staff, from the proximal environment, from shared equipment); or (2) the emergence of resistance, wherein the susceptible isolates that patients harbor acquire resistance mechanisms through mobile genetic elements (e.g., ESBL), or by expressing an MDR phenotype mediated by chromosomal genes due to certain stressors (e.g., AmpC) [34]. Preventing or curbing patient-to-patient transmission in human medicine is achieved through barrier precautions and infection control practices, e.g., hand hygiene, isolation precautions, cohorting with dedicated staff, environmental cleaning, surveillance programs to identify asymptomatic carriage, and sometime decolonization protocols whenever relevant [34]. This is also relevant to equine medicine, and has received attention mainly due to outbreaks with methicillin-resistant *Staphylococcus aureus* (MRSA), which have the potential to result in zoonotic transmission to veterinary personnel and pet owners [35]. In such an outbreak, which occurred a decade ago in our hospital, the strict implementation of many of these measures resulted in the cessation of the outbreak, and indeed six months after the intervention, both personnel and hospitalized horses were all MRSA-negative, and the intervention was considered successful [36]. In contrast, tackling the emergence of resistance requires the enforcing of adherence to AMS policies and programs, which is also relevant in equine medicine where the implementation of AMS is required, and indeed is evolving, although much more is required [32]. The fact that exposure to antibiotics was the only independent predictor associated with infection in general, and specifically with 3GCRE infection, implies that stewardship guidelines and practices are not yet sufficiently implemented. As depicted in the results, over 31% of the animals were admitted with 3GCRE infections from non-acute care settings, i.e., community-onset infections. This is not unexpected, since in a recent study, in the same veterinary hospital, on admission 19.6% of the horses were ESBL-PE shedders, and 20.8% of horses on farms were also ESBL-PE shedders [11]. This implies that AMS intervention, policies, monitoring and guidelines should be implemented in the community (horse farms and private practitioners) as well, in order to prevent the continued emergence and spread of resistances among animals (and humans). This study highlights again the importance of investing in AMS in veterinary medicine, specifically in community settings and farms.

There were multiple negative outcomes associated with 3GCRE infections in bivariate analyses (Table 2), as was previously reported in human studies [37]. Infections caused by 3GCRE were negatively associated with appropriate therapy administration and with the number of appropriate therapy days. In a recent human study, a delay in instituting appropriate therapy was an independent predictor for prolonged LOS, increased hospitalization costs, and mortality [38,39]. In our study, in multivariable separate models, 3GCRE was independently associated with a higher clinical failure rate, with surgery following the infection, and with 90-day mortality. This again emphasizes, as in the human studies [3], the epidemiological significance and relevance of 3GCRE infections in equine medicine.

Our findings reflect the complex molecular epidemiology and characteristics associated with 3GCRE infections among hospitalized equids. We have found a variety of bacterial species, i.e., 69% of samples were polymicrobial. In detailed molecular investigations, even the same bacteria which were analyzed belonged to multiple clones, including clones which were previously reported in equine isolates, e.g., *E. coli* sequence types (STs) 38, 361 [18] and 2179 [40], and *E. cloacae* STs 135 [41] and 254 [42]. Additional clones reported herein were previously reported among humans, but not among horses, i.e., *E. cloacae* STs 66 [43] and 182 [44], and *K. pneumoniae* STs 13, 35 [45], 528 [46] and 985 [47]. Some of these STs were identified as MDR international human clones. For example, *E. coli* ST38 is an emerging clone in Germany [48], *E. cloacae* ST66 was isolated from human hospitals in Japan, France, Spain and Israel [43], and *K. pneumoniae* ST35 was isolated from China and Yemen [49,50]. This ST dissemination has major implications for human medicine and the “one health” approach, due to the close human–horse proximities and interactions [51].

The most prevalent culture sites were the umbilicus and SSI. In human medicine, only a few reports describe 3GCRE umbilical infections [52,53,54]. In contrast, in neonatal foals, several studies have described or reported 3GCRE isolation from the umbilicus [16,18,55,56]. Umbilical remnant infections in foals can be successfully diagnosed and treated; however, they can also lead to potentially fatal complications by seeding bacteria to other parts of the body [57]. Umbilical remnant infection should always be considered in a foal with a patent urachus, which can be either acquired or congenital, and can act as an opening for bacterial invasion [57]. Care of the umbilical remnant and the environment in which the foal lives, the adequate passive transfer of immunity postpartum and intrauterine infection prepartum, are important factors in the development of umbilical remnant infection [57]. In terms of the pathophysiology, this resembles vertical transmission in humans, and highlights the fact that the neonate acquired the 3GCRE pathogen at birth (when labor occurred in outpatient settings), and not in healthcare. The management of umbilical infections is challenging among foals, due to the presumably low penetration of antibiotics into the infected tissue. This also promotes the emergence of resistance among offending isolates [55]. According to our knowledge, this is the first epidemiological investigation of 3GCRE infections among foals.

The study has several limitations. It is a retrospective chart-based study; therefore, some medical information may have been missing or incorrectly recorded. In addition, the study was conducted in a single center, and therefore the findings could not be generalizable automatically to other centers. The study also suffers from the small sample size of patients with 3GCRE infections; although the case–case–control design enabled us to increase somewhat its strength, many of the multivariable models were unstable, and this impacted the risk factors and outcomes analyses.

## 4. Materials and Methods

### 4.1. Study Design

A retrospective matched case–case–control investigation pertaining to horses and donkeys of all age groups was conducted at the Koret School of Veterinary Medicine, Veterinary Teaching Hospital (KSVM-VTH), Israel, from June 2017 to January 2019. KSVM-VTH is the only veterinary teaching hospital in Israel and has a large animal department that could contain up to 40 hospitalized horses. The study was approved by the Internal Research Committee of the KSVM-VTH, Israel (Protocol KSVM-VTH/15_2015). The investigation consisted of three groups of patients: (1) 3GCRE-infected patients, (2) 3GCSE-infected patients, and (3) uninfected control patients. Only the first 3GCRE for each patient was included in the final analysis (i.e., patient-unique cases). Resistant cases were defined as patients suffering from an infection (i.e., no asymptomatic carriers were included) due to an *Enterobacterales* spp., non-susceptible to ≥1 third-generation cephalosporines (e.g., ceftriaxone, ceftiofur, cefpodoxime, ceftazidime). Susceptible cases were defined as patients suffering from an infection caused by *Enterobacterales* spp., susceptible to all third-generation cephalosporins. The uninfected control group consisted of patients without any infectious syndrome, and with no *Enterobacterales* isolated. A 3GCSE case and an uninfected control were matched to each 3GCRE case (1:1:1 ratio). The matching criteria (in order of importance [23]) included the following: animal species (equine vs. assinus), bacterial species, age group (neonate/adult/geriatric), clinical syndrome, and time at risk (i.e., days from admission date to culture date). For uninfected controls, the time at risk was captured as the total length of stay [23]. A neonate was defined as an animal ≤30 days old [58] and a geriatric animal was defined as age ≥20 years [59]. The date of event was captured as the beginning of the first clinical sign or symptom of infection that was associated with the culture of interest. Appropriate therapy was defined as per the in vitro susceptibilities report given from 48 hours prior to the culture date and up to five days following the culture date [60]. Days to appropriate therapy were defined as the number of calendar days from culture to the first dose of "appropriate" therapy (as defined above). Data were extracted from medical records, including demographic data, recent exposures to health care environments and settings, background conditions, medical treatments, invasive procedures (in the past three months), empiric antibiotic regimens (i.e., from two days prior to culture date to three days following culture date), main antibiotic regimens (i.e., 3–14 days following culture date) and outcomes. Immunosuppression was defined as ≥1 of the following: neutropenia, or glucocorticoids/chemotherapy exposures in the previous three months. One-year mortality data were captured following a telephone interview with the owner.

### 4.2. Bacterial Isolates Collection, Identification and Susceptibility Testing 

All study isolates were subjected to Vitek-2 (BioMérieux, Inc., Marcy-l’Etoile, France) for species identification and phenotypic susceptibility testing (AST-N270 Vitek 2 card). Susceptibility to ofloxacin and imipenem was determined by using the disc diffusion assay (Oxoid, Basingstoke, UK). ESBL production testing was determined according to the Clinical and Laboratory Standards Institute (CLSI) benchmarks and guidelines [61]. Isolates were defined as MDR based on established criteria [24].

### 4.3. Molecular Characterization of ESBL-PE

Isolates were examined for the presence of the *bla*_CTX-M_ group by using a multiplex polymerase chain reaction (PCR) from ESBL-PE DNA lysates, as previously described [62]. Strains identified as *E. coli*, *K. pneumoniae* or *Enterobacter cloacae* were genotyped using an enterobacterial repetitive intergenic consensus (ERIC) PCR amplification using the following primer: 5-AAGTAAGTGACTGGGGTGAGCG-3’ [63]. Strains showing a distinct ERIC PCR pattern were further analyzed by MLST as previously described (IDGenomics, Seattle, WA, USA) [64,65,66].

### 4.4. Statistical Analyses 

Statistical analyses were performed using SPSS software (IBM; Version 24; SPSS Inc., Chicago, IL, USA). Data distribution was determined according to the skewness, kurtosis, and the Shapiro–Wilk’s test. Continuous variables were analyzed using t-tests or Mann–Whitney U-tests. Categorical variables were analyzed using the Fisher’s exact test or the Pearson chi-square test. In all analyses, *p* ≤ 0.05 indicated significance. Univariable and multivariable matched analyses determine the predictors of 3GCRE infection (vs. uninfected controls) and of 3GCSE infection (vs. uninfected controls). According to the case–case–control methodology, the eventual independent predictors of 3GCRE infection would be only those predictors associated with 3GCRE infection, but not with 3GCSE infection [23]. Logistic regression models were conducted by using the backwards stepwise method. Univariable and multivariable outcomes analyses (logistic regression) were conducted while enforcing the case type parameter (i.e., the 3GCRE group vs. the groups of 3GCSE and of the uninfected controls combined) in each outcome model.

## 5. Conclusions

This case–case–control study reveals and quantifies the clinical and epidemiological importance and significance of 3GCRE infections in equine medicine, and in equine hospitals. Larger studies in additional centers and countries are warranted. Antibiotic stewardship programs, both in hospitals and community settings, are mandatory in order to curb the continued dissemination and spread of 3GCRE pathogens.

## Figures and Tables

**Table 1:** Selected bivariable analyses comparing risk factors of patients infected with 3GCRE, patients infected with susceptible *Enterobacterales* and uninfected control patients (*n* = 29 in each group).

Parameter		3GCRE ^1^ No. (Valid % ^3^)	3GCSE ^2^ No. (Valid % ^3^)	Uninfected No. (Valid % ^1^)	3GCRE vs. Uninfected		3GCSE vs. Uninfected		3GCRE vs. 3GCSE	
					OR (95% CI)	*p*-Value	OR (95% CI)	*p*-Value	OR (95% CI)	*p*-Value
Demographics										
Age (Years), Median (Range)		2.25 (0–24)	3 (0–20)	3 (0–17)		0.93		0.756		0.786
Age Group	Neonates (<30 days)	12 (41.4)	12 (41.4)	12 (41.4)	1 (0.35–2.844)	>0.99	1.0 (0.352–2.844)	>0.99	1 (0.352–2.844)	>0.99
	Elderly (>20 years)	1 (3.4)	1 (3.4)	0	1.036 (0.97–1.11)	>0.99	1.036 (0.97–1.11)	>0.99	1 (0.06–16.791)	>0.99
Weight (Kg), Median (Range) /mean ±SD		125 (22–520)	118 (40–118)	200 (30–614)		0.859		0.94		0.92
Female Gender		19 (65.5)	21 (72.4)	12 (41.4)	0.372 (0.128–1.077)	0.065	0.269 (0.09–0.808)	**0.017**	1.382 (0.452–4.225)	0.57
Castrated Adult Male ^4^		2 (66.7)	3 (60)	6 (66.7)	1 (0.063–15.988)	>0.99	0.75 (0.078–7.21)	>0.99	1.333 (0.067–26.618)	>0.99
Pregnant Mare		4 (28.6)	5 (41.7)	2 (25)	1.2 (0.166–8.659)	>0.99	2.143 (0.299–15.355)	0.642	0.56 (0.11–2.8620	0.683
Shelter Resident		4 (13.8)	2 (6.9)	3 (10.3)	1.387 (0.282–6.83)	>0.99	0.642 (0.099–4.159)	>0.99	2.16 (0.363–12.84)	0.670
**Recent exposure to healthcare environments and/or settings**										
Recent Hospitalization (<3 months)		6 (20.7)	1 (3.4)	0 (0)	1.3 (1.053–1.605)	**0.023**	1.036 (0.967–1.109)	>0.99	7.304 (0.819–65.114)	0.102
Surgery Prior (<3 months) to the Date of Event ^5^		16 (55.2)	12 (42.9)	0 (0)	2.231 (1.49–3.34)	**<0.001**	1.75 (1.27–2.412)	**<0.001**	1.641 (0.576–4.675)	0.352
Urologic Procedure During Hospitalization, Prior to the Date of Event ^5^		14 (48.3)	11 (39.3)	2 (6.9)	12.6 (2.517–63.063)	**0.001**	8.735 (1.721–44.328)	**0.004**	1.442 (0.5.4–4.128)	0.494
Upper Airways Procedure During Hospitalization, Prior to the Date of Event ^5^		9 (31)	3 (10.7)	1 (3.4)	12.6 (1.476–107.543)	**0.005**	3.36 (0.328–34.415)	0.352	3.75 (0.895–15.715)	0.06
Plasma Therapy During Hospitalization, Prior to the Date of Event ^5^		10 (34.5)	4 (14.3)	0 (0)	1.526 (1.172–1.988)	**0.001**	1.167 (1.003–1.357)	0.052	3.281 (0.868–12.4)	0.116
Feeding/Nasogastric Tube During Hospitalization, Prior to the Date of Event ^5^		16 (57.1)	14 (46.4)	9 (32.1)	2.815 (0.946–8.376)	0.06	1.83 (0617–5.423)	0.247	1.538 (0.536–4.416)	0.422
Prior MDRO ^6^ Isolation (<1 year)		2 (6.9)	0 (0)	0 (0)	1.074 (0.973–1.186)	0.491	^a^	^a^	1.074 (0.973–1.186)	0.492
Prior ESBL Isolation (<1 year)		0 (0)	0 (0)	0 (0)	^a^	^a^	^a^	^a^	^a^	^a^
**Background conditions and co-morbidities prior to the date of event** **^3^**										
Chronic Lung Disease		2 (6.9)	3 (10.3)	0 (0)	1.074 (0.973–1.186)	0.491	1.115 (0.986–1.262)	0.237	0.642 (0.099–4.159)	>0.99
Neurologic Disease ^7^		6 (20.7)	1 (3.4)	4 (13.8)	1.63 (0.408–6.521)	0.487	0.223 (0.023–2.132)	0.352	7.304 (0.819–65.114)	0.102
Immunosuppression ^8^		7 (24.1)	1 (3.6)	1 (3.4)	8.909 (1.019–77.905)	0.052	1.037 (0.062–17.429)	>0.99	8.591 (0.981–75.221)	0.052
Hyperlactatemia ^9^		4 (66.7)	4 (40)	4 (36.4)	3.5 (0.431–28.447)	0.335	1.167 (0.2–6.805)	>0.99	3 (0.361–24.919)	0.608
Azotemia ^10^		7 (25.9)	4 (40)	10 (35.7)	0.63 (0.198–2.003)	0.432	0.3 (0.0815–1.113)	0.064	2.1 (0.537–8.217)	0.281
**Antimicrobial therapy prior (< 3 months) to the date of event ^5^**										
Any Antibiotic Treatment		28 (96.6)	20 (71.4)	3 (10.3)	242.667 (23.722–2482.349)	**<0.001**	21.667 (5.086–92.303)	**<0.001**	11.2 (1.296–96.787)	**0.012**
Penicillins		20 (71.4)	15 (53.6)	1 (3.4)	70 (8.1–604.917)	**<0.001**	32.308 (3.845–271.441)	**<0.001**	2.167 (0.717–6.55)	0.168
Fluoroquinolone		6 (21.4)	3 (11.1)	0 (0)	1.273 (1.049–1.544)	**0.01**	1.125 (0.985–1.285)	0.106	2.182 (0.486–9.796)	0.469
Aminoglycoside		23 (82.1)	9 (32.1)	1 (3.4)	128 (14.034–1182.052)	**<0.001**	13.263 (1.55–113.47)	**0.005**	9.711 (2.78–33.92)	**<0.001**
Polymyxin		6 (37.5)	9 (56.3)	0 (0)	1.6 (1.095–2.339)	**0.006**	2.286 (1.311–3.984)	**<0.001**	0.467 (0.113–1.92)	0.288
Metronidazole		4 (14.3)	5 (17.9)	0 (0)	1.167 (1.003–1.357)	0.052	1.217 (1.024–1.447)	**0.023**	0.767 (0.183–3.216)	>0.99
Cephalosporins		4 (14.3)	2 (7.1)	0 (0)	1.167 (1.003–1.357)	0.052	1.077 (0.972–1.193)	0.237	2.167 (0.363–12.922)	0.669
**Acute illness indices at the date of event ^5^**										
Sepsis ^11^		10 (34.5)	6 (20.7)	0 (0)	1.526 (1.172–1.988)	**0.001**	1.261 (1.047–1.518)	**0.023**	2.018 (0.62–6.569)	0.24

^1^ 3GCRE: Third-generation cephalosporin-resistant *Enterobacterales.* ^2^ 3GCSE: Third-generation cephalosporin-susceptible *Enterobacterales.* ^3^ Data are presented as valid percent, i.e., after removing the missing values from the denominator. ^4^ Only adult males included. Neonates were not included. ^5^ The date of event was defined as the date on which the first sign or symptom of the infection was documented, or the date of culture among patients with no sign or symptom documentation. ^6^ Isolates were defined as multidrug-resistant based on established criteria [24]. ^7^ Neurologic disease included any of the following: perinatal asphyxia syndrome, meningitis and radial nerve paralysis. ^8^ Immunosuppression was defined if one of the following criteria was positive: neutropenia on admission (neutrophil count < 2.9 cells/μL [25]), corticosteroids treatment (<1 month) or chemotherapy (<3 months). ^9^ Hyperlactatemia was defined as blood lactate levels >2.06 mmol/dL [26]. ^10^ Azotemia was defined as a baseline creatinine >1.9 mg/dL [27]. ^11^ Sepsis was defined based on established criteria [25,28]. ^a^ Analysis cannot be computed since at least one of the values is missing.

**Table 2:** Selected bivariable analyses comparing outcomes of patients with 3GCRE infection, patients with 3GCSE infections, and uninfected control patients (*n* = 29 in each group).

Parameter	3GCRE ^1^ No. (Valid % ^1^)	3GCSE ^2^ No. (Valid % ^3^)	Uninfected No. (Valid % ^1^)	3GCRE vs. Uninfected		3GCSE vs. Uninfected		3GCRE vs. 3GCSE	
				OR (95% CI)	*p*-Value	OR (95% CI)	*p*-Value	OR (95% CI)	*p*-Value
Total length of stay (LOS) after excluding the patients who died in hospital, days, median (range)	17.5 (2–181)	9 (2–59)	4 (2–17)		**<0.001**		**<0.001**		**0.027**
LOS from date of event ^4^ to discharge after excluding the patients who died in-hospital, days, median (range)	11.5 (1–181)	8 (0–59)	4 (2–17)		**0.002**		0.068		0.11
Additional hospitalization in the following 3 months	2 (10.5)	4 (17.4)	2 (8)	1.353 (0.173–10.592)	>0.99	2.421 (0.399–14.688)	0.407	0.559 (0.091–3.446)	0.673
Clinical failure ^5^	14 (51.9)	6 (21.4)	1 (3.4)	**30.3** **(3.57–250)**	**<0.001**	7.634 (0.855–66.667)	0.052	**3.77** **(1.157–12.195)**	**0.024**
Bacteriological cure ^10^	3 (60)	1 (50)		^a^	^a^	^a^	^a^	1.5 (0.055–40.633)	>0.99
Surgery following the date of event ^4^	18 (64.3)	15 (57.7)	7 (26.9)	**4.725** **(1.537–14.552)**	**0.005**	**3.231** **(1.081–9.656)**	**0.033**	1.463 (0.504–4.24)	0.483
Urologic procedure following the date of event ^4^	18 (62.1)	15 (57.7)	7 (26.9)	**4.295** **(1.42–12.997)**	**0.008**	**3.231** **(1.081–9.656)**	**0.033**	1.33 (0.466–3.792)	0.594
Upper airways procedures following the date of event ^4^	5 (17.2)	12 (41.4)	13 (44.8)	0.256 (0.076–0.86)	**0.045**	0.869 (0.307–2.458)	0.791	**0.295** **(0.088–0.994)**	**0.043**
Feeding tube/nasogastric tube following the date of event ^4^	16 (59.3)	15 (55.6)	25 (86.2)	0.233 (0.063–0.858)	**0.023**	**0.2** **(0.055–0.734)**	**0.011**	1.164 (0.395–3.425)	0.783
In hospital mortality	9 (31)	0 (10.3)	0 (0)	**1.45** **(1.136–1.851)**	**0.002**	1.083 (0.97–1.21)	0.49	3.9 (0.933–16.31)	0.052
14-days mortality ^6^	8 (30.8)	4 (16)	0 (0)	**1.444** **(1.118–1.866)**	**0.004**	1.19 (1.003–1.413)	0.11	2.333 (0.602–9.049)	0.214
90-days mortality ^6^	12 (48)	5 (20.8)	2 (8)	**8.462** **(1.61–44.53)**	**0.002**	3.026 (0.527–17.394)	0.247	3.257 (0.932–11.38)	0.059
1-year mortality ^6,7^	11 (47.8)	5 (20.8)	5 (18.5)	**4.062** **(1.166–14.154)**	**0.024**	1.158 (0.29–4.617)	>0.99	**3.508** **(0.996–12.359)**	**0.046**
Appropriate therapy ^8^ (given 2 days before to 5 days after culture date)	3 (11.5)	19 (76)		^a^	^a^	^a^	^a^	**0.041** **(0.009–0.187)**	**<0.001**
Days of appropriate therapy ^8^, median (range)/mean ± SD	0 (0–16)	7.1±5.9		^a^	^a^	^a^	^a^		**<0.001**
Days to appropriate therapy ^8^, median (range)	0 (0–1)	0 (0–5)		^a^	^a^	^a^	^a^		0.929

^1^ 3GCRE: Third-generation cephalosporin-resistant *Enterobacterales.* ^2^ 3GCSE: Third-generation cephalosporin-susceptible *Enterobacterales.* ^3^ Data are presented as valid percent, i.e., after removing the missing values from the denominator. ^4^ The date of event was captured as the beginning of the first clinical sign or symptom which defines infection, which was associated with the culture of interest. ^5^ Clinical failure was defined as non-recovery (for infections, non-recovery from infectious syndrome; for uninfected, non-recovery from the disease leading to hospitalization). ^6^ Mortality from culture date. ^7^ One-year mortality data were captured following a telephone interview with the owner. ^8^ Appropriate therapy was defined according to in vitro susceptibilities (of the microbiology lab report). ^a^ Analysis cannot be computed since at least one of the values is missing.

## Data Availability

Data is contained within the article or Supplementary Material.

## Supplementary Materials

The following are available online at https://www.mdpi.com/2079-6382/10/2/155/s1. Table S1: Descriptive statistics for the entire study population (*n* = 1620 horses and donkeys).
