# Supplementary material for: Third Generation Cephalosporin Resistant Enterobacterales Infections in Hospitalized Horses and Donkeys: A Case–Case–Control Analysis
Source: Antibiotics (Basel). 2021 Feb 4;10(2):155. doi: 10.3390/antibiotics10020155 (PMC7913880; doi:10.3390/antibiotics10020155)
Supplement: Supplementary file 1 [file antibiotics-10-00155-s001.pdf]

antibiotics

MDPI

Table S1. Descriptive statistics for the entire study population (*n* = 1620 horses and donkeys).

Parameter	Classification	Valid Percentage % (No. of Cases)
Sex	Female	58 (*n* = 935/1612)
	Male	42 (*n* = 677/1612)
Horse Breed	Arabian	43.8 (*n* = 412/940)
	Local breeds	30 (*n* = 281/940)
	Gaited horses (Missouri fox trotter, Rocky mountain horse, Single foot, Tennessee walking horse,)	8.2 (*n* = 77/940)
	American Quarter horse	6 (57/940)
	Warmblood	3.9 (*n* = 37/940)
	Friesian	2.4 (*n* = 23/940)
	Thoroughbred	1.6 (*n* = 15/940)
	Others	4.1 (*n* = 38/940)
Median Age on Hospital Admission, Years (range)		5 (0–27)
Age Groups	Neonates (0–30 days)	14.8 (*n* = 239/1620)
	Weanling (30 days- 1 year)	11.6 (*n* = 188/1620)
	1–3 years	17.1 (*n* = 277/1620)
	Adult (3–20 years)	55 (*n* = 891/1620)
	Geriatric (>20 years)	1.5 (25/1620)
Median Hospital Length of Stay, Days (range)		3 (0–190)
